# Challenges on Diagnoses and Assessments Related to Autism Spectrum Disorder in Brazil: A Systematic Review

**DOI:** 10.3389/fneur.2021.598073

**Published:** 2022-01-20

**Authors:** Ricardo Sukiennik, Josemar Marchezan, Francisco Scornavacca

**Affiliations:** ^1^Pediatrics Department, Universidade Federal de Ciências da Saúde de Porto Alegre (UFCSPA), Porto Alegre, Brazil; ^2^Postgraduate Program in Medical Sciences, Universidade do Vale do Taquari (Univates), Lajeado, Brazil

**Keywords:** austim, ASD, autismo spectrum disorders, diagnoses, diagnoses tools

## Abstract

Being a continental country, with over 210 million citizens, Brazil is similar to all of those who are part of the LAMIC (Low and middle income countries). It shows a big concentration of wealth, mainly in its south and southeast regions, as well as areas with immense poverty. In that sense, the health system also faces a huge amount of contrast. Inside University hospitals and facilities there are sophisticated tools and trained doctors prepared to assist in any kind of medical subject, including autism. But, unfortunately, at other times, the access to a good health system is made much harder. This results in many issues in the medical community, e.g., looking at the data regarding autism, there is a high average of the age of diagnosis. Another issue is the low number of professionals trained in ASD diagnosis and the few tools translated to Portuguese.

## Introduction

Brazil is a continental country and is the fifth largest country in the world in territorial extension with over 210 million citizens (1, 2). Brazil is divided into five regions with different geographical, demographic, cultural, and financial characteristics (3), including different health indicators (4). Similar to other low- and middle-income countries (LMICs), Brazil shows a great concentration of wealth as well as areas with immense poverty. In this sense, access to the health system is also a great contrast (5).

In Brazil, the Single National Health System (SUS) (*Sistema Único de Saúde* in Portuguese) provides access to health services for all citizens. This system is organized regionally and is composed of services with different degrees of complexity. It is also financed and coordinated by various government agencies (6–8). The public health system is not always able to offer excellent care for the entire population, and the private health system only services 20–45% of the citizens. Some hospitals and clinics attend both systems, but, in certain fields, the private system is more complete with tools, experts, and resources that are not present in the public system (9, 10).

Autism spectrum disorder (ASD) is a complex neurobiological development disorder characterized by two main behavioral components: (1) difficulty with communication and social interactions and (2) restrictive and repetitive behaviors, interests, and activities (11). ASD is a disorder that manifests with a wide variety of symptoms in the cognitive, emotional, and neurobehavioral areas. Although the characteristics that compose it are well-defined, the great heterogeneity of findings in each individual makes its recognition challenging. The disorder encompasses extremely heterogeneous phenotypes, especially in the mildest cases of the spectrum, and the severity of central deficits varies greatly between patients (12–15).

The current prevalence points out that one in 54 children aged 8 years has ASD in the USA, and the ratio is higher in boys than in girls (4:1) (16). The prevalence of ASD makes it one of the most frequent neurological development disorders, representing a major public health concern, and it leads to high social and economic costs (17). There are no Brazilian studies that reliably estimate the prevalence of ASD; therefore, the federal government included the pathology in the 2020 national census (18). Current estimates show that about 1.5 million people have ASD in Brazil (15, 19, 20). A study analyzing the profile of children attended at the Child and Adolescent Psychosocial Care Center (Centros de Atenção Psicossocial Infanto-Juvenil [CAPSi]), from 2008 to 2012, showed that 23.6% of a total of 837,068 visits were related to developmental disorders (21).

In the absence of a biological marker, the diagnosis of autism remains a clinical decision (14, 22, 23), and instruments and scales are often used to aid in the diagnosis (14). Even in developed countries, studies describe the difficulty of early diagnosis, the parents' pilgrimage in different health services, and their discontentment with the diagnostic process (24–27). In addition, factors such as family income, residence in rural areas, ethnicity, child impairment, clinical presentation, and parental concern about initial symptoms are associated with later diagnosis (28–30). Therefore, it is expected that LMICs will have even greater difficulties in early diagnosis.

The diagnosis of ASD is based on a qualitative assessment of behavioral patterns and is directly influenced by the complexity and variability in the presentation of the disorder (e.g., levels of severity, association with intellectual disability, and other medical conditions). These characteristics have led to the development of a significant number of international instruments focusing on identification and early diagnosis (31, 32). Experience from high-income countries suggests that incorporating screening tools into routine healthcare visits can result in earlier and more accurate identification of children with developmental disorders, compared to only relying on clinical impressions (33). The use of ASD screening and diagnostic instruments in Brazil is still limited, representing an obstacle to the expansion of research in this field and to the improvement in the quality of health services. Although some instruments have been translated and validated, critical examination of the psychometric quality of these studies is still lacking in Brazilian publications (31).

## Objectives

We aim to identify all data related to the tools and identification process of children and adolescents with ASD in Brazil.

## Review Question

What data do we have related to the tools and the process of identifying children and adolescents with ASD in Brazil?

## Methods

We searched in PubMed (maintained by the United States National Library of Medicine at the National Institutes of Health), Scientific Electronic Library Online (SciELO), and Literatura Latino-Americana e do Caribe em Ciências da Saúde (LILACS) for systematic reviews about the tools and identification process for children with ASD in Brazil, and one article of 2010 was found. The authors performed a systematic review of the literature on the diagnosis of ASD in Brazil. The review follows the Preferred Reporting Items for Systematic Reviews and Meta-Analyses (PRISMA) system of reporting (34). In regard to the article and abstract selection, the following inclusion criteria were used: (1) articles and abstracts published in the last 20 years; (2) articles that have at least one Brazilian researcher listed as an author; and (3) studies focused in identification/diagnosis of ASD.

The exclusion criteria included papers published more than 20 years ago, with no Brazilian authors listed, and whose languages were English and Portuguese. They also included articles of review, neurobiological and genetic bases, ASD comorbidities, epidemiological studies, phenotype and endophenotype studies, and intervention trials.

The articles were identified through a research of the major biomedical literature databases: PubMed, SciELO, and LILACS. The following research terms were used, alone and in combination: “autism,” “ASD,” and autism spectrum disorder [Mesh]; “assessment,” “diagnostic criteria,” and diagnosis [Mesh]; “scale,” “instrument,” “tool,” and “Brazil” [Mesh]; and “Brazilian.” An eligibility assessment was performed independently in an unblinded standardized manner by three authors. Disagreements between reviewers were resolved by consensus.

## Results

**Figure 1:**
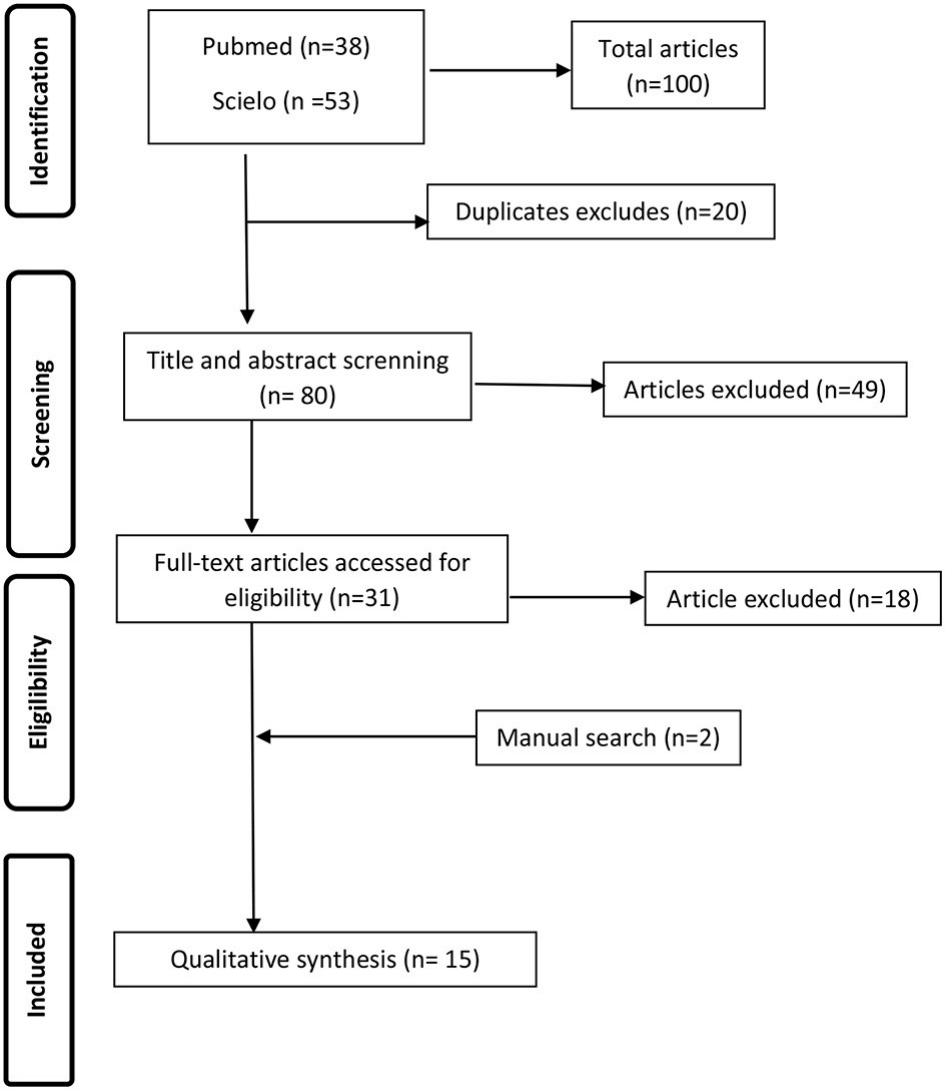
Article selection.

After a systematic review, 15 articles were identified in the last 20 years (Figure 1). These articles are summarized in Table 1. We found seven validation studies, one before–after study, and eight cross-sectional studies.

**Table 1:** Selected articles.

**References**	**Year**	**Title**	**Study**	**Finding/Summary**
Barbosa et al. (35)	2015	Propriedades psicométricas da Escala de Responsividade Social 2 para Transtornos do Espectro Autista	Translation/validation	ERS-2 Portuguese version can be used as a screening tool; however, some items were not statistically consistent, especially those related to mild ASD.
Becker et al. (36)	2012	Tradução e validação da ADI-R (Autism Diagnostic Interview-Revised) para diagnóstico de autismo no Brasil	Translation/validation	Translated and validated by the ADI-R scale for Brazilian Portuguese.
Bordini et al. (6)	2014	Impact of training in autism for primary care providers: a pilot study	Before–after trial	The trained providers significantly improved their ASD knowledge after training in comparison with pre-training. Clinical practice also changed: 4 months after the training program, the providers had referred six times as many suspected cases of ASD to a specialized mental health service in comparison with the previous 4 months.
Losapio and Pondé (37)	2008	Tradução para o português da escala M-CHAT para rastreamento precoce de autismo	Translation/validation	Translated and validated by the M-CHAT scale for Brazilian Portuguese.
Machado et al. (38)	2016	Respostas parentais aos sinais clássicos de autismo em dois instrumentos de rastreamento	Cross-sectional study	Isolated points of the instruments Questionário de Indicadores de Risco para o Desenvolvimento Infantil (IRDI) and M-CHAT were unable to predict ASD in relation to the set of questions.
Machado et al. (19)	2016	Appropriateness of using autism spectrum disorders screening tools in a hearing evaluation service	Cross-sectional study	It assessed ASD signs in children referred to an audiological center to investigate hearing loss. Only 18% of the 43 children assessed had hearing loss, while 60% had ASD signs.
Marques and Bosa (39)	2015	Protocolo de Avaliação de Crianças com Autismo: Evidências de Validade de Critério	Cross-sectional study	Preliminary assessment of Protocolo de Avaliação para Crianças com Suspeita de Transtornos do Espectro do Autismo (PRO-TEA), suggesting that this instrument may assist in the ASD diagnosis.
Marteleto et al. (40)	2008	Administration of the Autism Behavior Checklist: agreement between parents and professionals' observations in two intervention contexts	Cross-sectional study	Verified the discrepancy in the responses in the Autism Behavior Checklist of parents and therapists of ASD children.
Duarte et al. (41)	2003	The CBCL and the identification of children with autism and related conditions in Brazil: pilot findings	Cross-sectional study	The Child Behavior Checklist (CBCL) scale was applied to ASD children, children with other psychiatric disorders, and healthy children. Scores on the “Thought Problems” and “Autistic/Bizarre” scales were related to cases of autism.
Galdino et al. (42)	2018	Evidence of validity of the Autism Mental Status Examination (AMSE) in a Brazilian sample	Cross-sectional study	The data suggest that this tool can be used for the screening of ASD.
Marteleto and Pedromônico (43)	2005	Validity of Autism Behavior Checklist (ABC): preliminary study	Translation/validation	It is a promising tool for identifying children with autism, especially with a cutoff point of 49.
Pacifico et al. (44)	2019	Preliminary evidence of the validity process of the Autism Diagnostic Observation Schedule (ADOS): translation, crosscultural adaptation and semantic equivalence of the Brazilian Portuguese version	Translation/validation	Translated and validated by the ADOS scale for Brazilian Portuguese.
Pereira et al. (45)	2008	Childhood autism: translation and validation of the Childhood Autism Rating Scale for use in Brazil	Translation/validation	Translated and validated the CARS into Brazilian Portuguese.
Ribeiro et al. (46)	2017	Barriers to early identification of autism in Brazil	Cross-sectional study	Family members of patients with ASD, describe the difficulties and delay in diagnosis.
Sanvicente-Vieira et al. (47)	2013	Revised Reading the Mind in the Eyes Test (RMET)—Brazilian version	Translation/validation	Translated and validated by the RMET scale, in both paper-and-pencil and computerized versions. The RMET is a well-accepted instrument for the assessment of theory of mind, an important component of social cognition.

Most studies are focused on the validation of scales and instruments and on the correlation of these with the diagnosis of ASD. Two studies stand out for us. The first led by Ribeiro et al. (46), which focuses on the barriers to early identification of autism in Brazil. This study shows us that the distance between the suspected diagnosis of ASD by parents and the formal diagnosis of the disease had an average delay of 3 years. Most mothers described their interactions with the doctorsas negative, and they felt discouraged to express their concerns again. The second was that of Bordini et al. (6), which shows that the training of health workers significantly increases knowledge about ASD. The number of patients referred to specialized ASD treatment centers has increased sixfold in just 4 months after training.

## Discussion

After the systematic analysis, it was found that there were few data on the TEA diagnosis process in Brazil and that most of the articles are related to the topic of translation processes and validation tools for Brazilian Portuguese.

It draws our attention to the fact that most of the difficulties encountered are late diagnosis, lack of training of health teams, problems of doctor–patient relationship, lack of knowledge about ASD, access to the health system, high cost of training professionals, and the high cost of tools for assessment of ASD patients. These problems are described in studies in other LMICs as can be seen in the paper of Ha et al. and blunt commentary of Ha et al. (48) and Durkin et al. (49).

Although the Brazilian mental health system is fully integrated with SUS (8, 50), unequal distribution of resources among different regions of Brazil and high levels of individual income inequality make access to care a significant challenge for many children and adolescents with mental health problems (7), the majority being ASD children who are not receiving specialized treatment (6).

There is a concentration of experts and services on some university sites, mainly in some south and southeast regions, which are the richest regions of the country. In addition, more than half of the doctors are concentrated in state capitals, where less than a quarter of the country's population lives (51). Inside universities and large hospitals, there are sophisticated tools and trained teams prepared to assist with any kind of medical diagnosis, including ASD. Unfortunately, access to the health system is made much harder in other situations. Some poor areas do not have trained and structured services to identify developmental delays, including ASD (7). It is estimated that only one in five children or adolescents in Brazil receive adequate mental care due to a shortage of specialized services, especially in the country's north and midwest regions (21, 52, 53).

The lack of professionals trained to recognize the manifestations of the disorder and the shortage of specialized services are associated with late ASD diagnosis (24). Even though the public health system should officially identify children with a developmental delay, some children (mainly the poorest) do not have access to skilled health services. Mandell et al. (54) showed that white children are diagnosed at 6.3 years old, while African American children are diagnosed at 7.9 years old, on average in the USA. These racial and ethnic differences in the age of diagnosis may be related to institutional factors, such as difficulties in a family's access to health services (30), although there are no similar studies in the Brazilian population. Based on other studies (55, 56), we believe that there is a similar reality in Brazil.

Although parents had concerns about their children's development, they had some obstacles until the diagnosis. Ribeiro et al., in an interesting work on the barriers of ASD diagnosis in Brazil, described the negative experiences of family members (56.3%) when reporting symptoms of autism to pediatricians and feeling discouraged to express their concerns again. Parents sometimes hear phrases from health professionals like “children should not be compared to each other” and “boys have a slower development rate” or they “are more agitated than girls” (46). Similar findings were reported in the UK and France, showing high rates of discontent with the diagnosis process. Even with parents suspecting that something was wrong with their children, it is not unusual for pediatricians to instruct them not to worry and to wait (26, 27). In Brazil, cases of 3- to 4-year-old children with a speech delay who are referenced to speech therapy without screening for ASD still occur. Interestingly, Machado et al. while evaluating the presence of signs of ASD in children referred to a hearing center to investigate hearing loss, despite the small sample, found signs for ASD (60%) more often than hearing loss (18%) (19).

Delays in obtaining an ASD diagnosis contribute to parental distress and postpone the start of therapeutic intervention, which, in turn, may affect the patients' long-term functional outcome and social adaptation (6, 25, 57, 58). Some advances have occurred, such as the promotion of early diagnosis by the Brazilian Academy of Pediatrics (59) and the release of guidelines for ASD diagnosis by the Brazilian Ministry of Health (60, 61). Unfortunately, validated protocols or screening algorithms for early ASD detection have not been implemented in most Brazilian public health facilities (15, 19, 20, 60), in spite of the World Health Organization (WHO) recommendation that LMICs should have an early ASD detection program (62). The insufficient information and clinical training of primary healthcare professionals, including scarce teaching about autism in medical schools, also contributes to the problem (6, 19, 63).

Despite the relevance of this topic, the number of Brazilian scientific publications on the care of children with ASD from the perspective of their family members is still scarce (46, 64, 65), and the few existing studies have a small number of participants. It is noteworthy that there are few studies focused on the training of health professionals to identify children with ASD in a clinical practice, and there is also a lack of initiatives to guide education workers, such as kindergarten educators, to identify these children. Perhaps these initiatives are one of the keys to early identification of children with ASD.

Diagnosing ASD is a challenging task (66). The screening tools help to identify children who may have developmental delays, allowing their early referral to specialized centers. Some screening tools are used primarily in pediatric practices, while others are used by school systems or in other community settings. Diagnostic tools, although they cannot be used as a basis (the diagnostic process should include information from parents/caregivers and child observation and interaction along with the usage of clinical judgement), aid in diagnosis (38, 66–68).

There is a lack of consensus on which screening tools are most effective especially when the tools are used in cultures other than those in which they were developed, which occurs often in LMICs. Routine screening is an important first step toward addressing the need for services in LMICs, but high-quality tools take time to be conceptualized, developed, piloted, and validated, before implementation can happen (33). Most tools that help the diagnosis of autism are developed in English and need to be translated and validated for use in clinical practice in Brazil. We found many studies that made this process. Despite the success demonstrated in some papers, few tools were fully tested, and there is a great delay between their development and their validation for use in Brazil. As an example, Losapio and Pondé (37) translated the M-CHAT, a screening tool, in 2008, but the original article was published in 2001 (37, 69). Likewise, the CARS, which is so important in helping the identification of ASD children, was validated in the same year (2008) (45), 20 years after the original paper was published (70). In addition, health professionals make little use of these instruments in daily practice. Many parents of ASD children report that they visited many different health professionals in search of a diagnosis during their child's early years, but no specific ASD screening was performed (19, 71).

There is a lack of validated/translated tools to identify children with ASD in clinical practice, resulting in some initiatives to develop new tools that could be used in Brazil. However, this kind of work demands time and has obstacles, as we see in the work of Bosa et al. (72).

In summary, the difficulties encountered in Brazil do not seem to be very different from those encountered in other developing countries. According to Stewart and Lee (73), community-based screening was shown to be an effective method for identifying ASD in communities with limited clinical resources, and these studies offer the opportunity to identify individuals with symptoms across a wider spectrum.

Access to healthcare providers who are capable of diagnosing and treating individuals with ASD can be very limited in LMICs (73). An alternative to improve this situation is to invest in training primary healthcare workers and non-specialists. The WHO is conducting an alternative for the early intervention of children with ASD. This initiative is named the WHO Caregiver Skills Training (WHO CST) program and is designed to train people who are not specialists in the health field to perform health interventions aimed at delayed development in children, including ASD. This program is designed to use a combination of group sessions (e.g., community centers) and individual sessions in children's homes. The group session is tailored to teach the caregivers to carry out the necessary interventions for children with developmental disorders, while keeping the costs low. The session at home is held to adapt interventions to the individual needs of each child and family (74). This type of intervention is based on reviews that demonstrate that interventions performed by caregivers guided by non-specialists with ASD patients are effective (75, 76). The WHO CST program is currently undergoing field testing in more than 30 countries in regions throughout the world (77). Randomized studies are being carried out in Pakistan and Italy, and cultural adaptations are also being carried out for each community when necessary (74, 77–79).

## Data Availability Statement

The raw data supporting the conclusions of this article will be made available by the authors, without undue reservation.

## Author Contributions

All authors listed above participated independently in the selection of articles and review of the topic. After an initial selection, the content was reviewed together and the article presented here was elaborated. All authors contributed to the article and approved the submitted version.

## Conflict of Interest

The authors declare that the research was conducted in the absence of any commercial or financial relationships that could be construed as a potential conflict of interest.

## Publisher's Note

All claims expressed in this article are solely those of the authors and do not necessarily represent those of their affiliated organizations, or those of the publisher, the editors and the reviewers. Any product that may be evaluated in this article, or claim that may be made by its manufacturer, is not guaranteed or endorsed by the publisher.
